# Are Chlamydia Trachomatis and Neisseria Gonorrhoeae Screenings in Pregnant Women Being Properly Performed? A Single-Center Retrospective Observational Study in Italy

**DOI:** 10.3390/pathogens13070570

**Published:** 2024-07-08

**Authors:** Vito Mondì, Jacopo Caravetta, Piermichele Paolillo, Nicola Salce, Chryssoula Tzialla, Barbara Vasapollo, Herbert Valensise, Manuela Bedetta, Simonetta Picone

**Affiliations:** 1 Neonatology and Neonatal Intensive Care Unit, Policlinico Casilino, Via Casilina 1049, 00169 Rome, Italy; jcaravetta.polcas@eurosanita.it (J.C.); pmpaolillo.polcas@eurosanita.it (P.P.); nsalce.polcas@eurosanita.it (N.S.); mbedetta.polcas@eurosanita.it (M.B.); spicone.polcas@eurosanita.it (S.P.); 2 Neonatal ad Pediatric Unit, Polo Ospedaliero Oltrepò, ASST Pavia, Via Volturno 14, 27058 Voghera, Italy; chryssoula.tzialla@unipv.it; 3 Department of Surgical Sciences, University of Rome “Tor Vergata”, Via Montpellier 1, 00133 Rome, Italy; barbara.vasapollo@hotmail.it (B.V.); valensise@uniroma2.it (H.V.); 4 Obstetrics and Gynecolocy Unit, Policlinico Casilino, Via Casilina 1049, 00169 Rome, Italy

**Keywords:** *Chlamydia* trachomatis, neisseria gonorrhoeae, pregnant, screening

## Abstract

A new Italian intersociety position statement on the prevention of ophthalmia neonatorum was published in 2023. In this document, attention was paid to the indications for the screening of gonococcal and chlamydial infections during pregnancy according to the international and national guidelines for the prevention of sexually transmitted infections (STIs). We conducted an observational retrospective study to assess whether the current guidelines for the prevention of STIs are being followed correctly. From February to August 2022, 2507 women nearing childbirth were enrolled. Among them, 42.4% received a swab for *Chlamydia* and only 0.5% for gonococcus. Concerning the geographical area of origin, most of the screened women came from Western Europe. None of the women who received gonococcal swabs and only 105 women out of 1062 screened for *Chlamydia* were under 25 years of age. Overall, only seven swabs were positive for *Chlamydia*, while none were positive for gonococcus. Concerning the age, geographical area of origin, and medical history of the women with a positive screening for *Chlamydia*, all were over 25 years old, six were from Western Europe, one was from South America, and none had other STIs. Although monocentric in nature, this study shows that the guidelines are not being followed correctly.

## 1. Introduction

In 2023, a new intersociety document on the prevention of ophthalmia neonatorum (ON) was issued in Italy [1]. ON is a conjunctivitis that occurs in the first month of life and affects 1.6 to 12% of all newborns [2,3,4]. In the past, ON was exclusively associated with eye infection due to *Neisseria gonorrhoeae* (NG) [5]. Currently, several bacteria are implicated in this disease. NG now accounts for <1% of the reported cases of ON in the United States, while cases due to *Chlamydia trachomatis* (CT) range from 2% to 40%. Other bacteria, such as *Staphylococcus* spp., *Streptococcus* spp., *Haemophilus* spp., and other Gram-negative bacterial species, account for 30% to 50% of cases [6]. ON is generally a mild disease. The most severe forms of conjunctivitis appear to be caused by NG and CT. Gonococcal ophthalmia occurs in 30% to 50% of infants exposed during birth and born to positive untreated women. In these cases, the disease can rapidly progress to corneal ulceration, globe perforation, and permanent visual impairment [7]. Infants born to women with untreated chlamydia infection at delivery have a 50% risk of acquiring CT, a 30% to 50% risk of developing neonatal conjunctivitis, and a 10% to 20% risk of developing pneumonia [8]. Untreated chlamydial conjunctivitis can be associated with corneal and conjunctival scarring, hemorrhagic conjunctivitis, and loss of vision [9,10,11].

CT and NG are the two most common bacterial sexually transmitted infections (STIs), and they are a major public health concern. When an infected woman is left untreated, vertical transmission can occur at the time of delivery.

In the new intersociety document [1], attention was paid to two different objectives: neonatal ocular prophylaxis and maternal screening. In assessing the need for universal neonatal prophylaxis, prevention strategies used in other states were also analyzed. There is considerable global variability in recommendations on whether to use ophthalmia neonatorum prophylaxis, and the proper agent used [12]. Universal neonatal ocular prophylaxis was discontinued in the United Kingdom in the 1950s and in Australia, Denmark, Norway, and Sweden in the 1980s [13]. As of 2015 (reaffirmed in 2021), the Canadian Pediatric Society no longer recommends routine neonatal prophylaxis [14]. Universal prophylaxis with 0.5% erythromycin ointment is currently recommended in the United States [15]. Through a survey conducted in Italy [16], it was found that prophylaxis was being performed with drugs not recommended by the World Health Organization [17,18]. According to the new intersociety position statement, in Italy, prophylaxis is no longer recommended for all infants at birth, except for those born from unintended pregnancies, mothers at risk of STIs, and mothers from areas with a high prevalence of gonococcal and chlamydial infection [1].

In creating this document, the importance of maternal screening during pregnancy was highlighted by considering the risk factors identified by the Italian Institute of Health (ISS, Istituto Superiore della Sanità), listed in the new guidelines on physiological pregnancy (Table 1) [19].

This retrospective study aimed to evaluate whether the criteria discussed during the writing of the statement were already pre-considered, and therefore whether there was a need for greater awareness of the necessity of adequate maternal screening in the prevention of ON.

## 2. Materials and Methods

This was a retrospective observational study. All the women near childbirth at term (gestational age (GA) of more than 37 weeks) admitted to the “Policlinico Casilino” hospital from February to August 2022 were enrolled.

The following data were collected anonymously for each woman: age, nationality (country of birth), positivity for sexually transmitted diseases (syphilis, hepatitis B and C, HIV), GA, mode of delivery, and time of rupture of membranes (pROM). Regarding the screening swabs for CT and NG, whether they were performed or not performed and, if performed, whether they were positive or negative was recorded. Data in the medical records refer to investigations not necessarily prescribed by gynecologists working in our hospital. In our study, we considered swabs performed in the third trimester, the only ones noted in the medical records. According to the new ISS guidelines [19], women with risk factors must be swabbed in the first and third trimester, independently of the result of the first trimester swabs. No criteria were specified for which screening was necessary, but it was assessed whether they were evaluated according to the risk factors identified by the ISS [19]. In addition, in women with a positive swab, it was checked whether antibiotic therapy was performed. Neonates born to women with a CT positive swab were followed up in the first two weeks of life to monitor the onset of ON. Neonates born to women with an NG positive swab, whether untreated or inadequately treated, were evaluated by swab and treated.

In the statistical analysis, we compared general data (GA, pROM, type of delivery) of the women included in the study to assess whether there were significant differences in the populations that had or had not been swabbed. Afterward, we evaluated the risk factors included in the new intersociety document and the ISS guidelines. Specifically, it was evaluated whether there was a difference in the number of women who had or had not been screened for CT and NG in relation to their nationality, age, and IST. Finally, it was assessed whether there was a difference in the number of women who had a negative or positive swab for CT or NG in relation to age and STI.

Concerning the statistical analysis, continuous variables were presented as the mean and standard deviation and compared with the Student’s t test. When using a non-parametric test, stratified data were compared with Fisher’s exact test or the Mann–Whitney test, as appropriate. A *p*-value of ≤0.05 was considered significant. The analysis was performed using SPSS version 20.0.0 (Statistical Analysis System, Chicago, IL, USA).

## 3. Results

From February to August 2022, 2507 women were enrolled in our study.

### 3.1. General Data

The median maternal age was 33.6 years. The mean GA at birth was 39.2 weeks.

The majority (86%) of admitted women had citizenship of a Western European country. The geographical distribution of countries is specified in Supplementary File S1. Concerning screening tests, 1455 women (57.6%) had not been tested for CT. Only 13 women (0.5%) received an NG swab (Table 2). All women who had been screened for NG had also been screened for CT. The identification of NG and CT on swabs is based on nucleic acid amplification tests (NAATs).

Overall, only 10 women were positive for STIs (corresponding to 0.6% of the total sample). Specifically, three were positive for syphilis, six for HBV, and one for HIV.

### 3.2. Screening in Different Populations

When assessing the differences in age, the timing of membrane rupture, and GA between the women who were screened for CT and NG, the only significant difference was in the GA in the group that underwent CT screening (*p* = 0.05) (Table 3). Comparing the delivery type in the different groups, there were no significant differences (Table 4). Analyzing the nationality of the mother (Table 5) and comparing the origin of women who had been screened for CT, there were no significant differences in the geographical distribution. When evaluating the NG swabs, most of the screened women were from Western Europe (11 out of 13 screened women). The difference in the geographical population’s distribution of women based on those who received NG swabs was significant (*p* < 0.001).

### 3.3. Screening Related to Age and STIs

Among the STI-positive women, none were screened for NG and only four (one HIV-positive and 3 HBV-positive) for CT: all four women were negative for CT. On the contrary, all women tested for NG were unaffected by STIs and none of the seven CT-positive women had an STI.

Regarding age, the prevalence of women screened was over 25 years of age. In the group of women aged under 25, only 57 out of 162 were screened for CT (*p* = 0.05), and none for NG. All 13 women screened for NG (all with negative results) were older than 25 years. Concerning CT swabs, only one woman under 25 years and 6 over 25 years (Table 6 and Table 7) tested positive. In Table 7, the NG swab results in relation to STIs were not included because no women with STIs had undergone NG screening. Hepatitis C was also not included because no women were recorded as positive for HCV.

### 3.4. Ophthalmia Neonatorum

In our unit, in the first 7–10 days of life, all newborns were routinely evaluated in the outpatient services, regardless of the maternal screening results. Neonates born to women with CT-positive swabs were followed up in the first two weeks of life. No cases of ON were reported during this period despite CT-positive swabs.

## 4. Discussion

Globally, STIs remain a challenging public health concern. Chlamydial and gonococcal infections are among the most common STIs around the world.

### 4.1. Chlamydial and Gonococcal Infection in Pregnant Women

The anatomy of women increases their susceptibility to chlamydial and gonococcal infections [20]. Female urogenital anatomy is more exposed and vulnerable to STIs compared to male urogenital anatomy, particularly because the vaginal mucosa is thin, delicate, and easily penetrated by infectious agents [21]. The impact of vertical transmission cannot be ignored [22]. Most cases of STIs are asymptomatic, which leads to the persistence of the infection, and in some cases, the development of complications that may be irreversible or even fatal. In addition to ON, infertility, ectopic pregnancy, premature rupture of membranes, low-birth-weight infants, and preterm delivery have been reported in women of childbearing age and during pregnancy [23,24].

Therefore, as most NG and CT infections are asymptomatic, screening tests are useful for prompt diagnosis [25,26]. Chlamydial and gonococcal infections are diagnosed by detecting these bacteria in urogenital, rectal, oropharyngeal, or ocular secretions [27]. According to the European and CDC guidelines, the detection of CT and NG should be performed using NAATs, which are based on the amplification of the specific bacteria nucleic acid target sequences and are mostly used due to their higher sensitivity [28,29,30].

Gonococcal and chlamydial infections are treated exclusively with antibiotics. In adolescents and adults, the CDC guidelines for the treatment of CT recommend doxycycline 100 mg orally, twice daily, for 7 days [31]. Doxycycline is contraindicated during the second and third trimesters of pregnancy because of the risk of tooth discoloration. Human data have revealed that levofloxacin, even if it presents a low risk for the fetus during pregnancy, is potentially toxic. Conversely, data from animal studies have raised concerns regarding cartilage damage to neonates [32,33,34]. Clinical experience and published studies indicate that azithromycin is safe and effective during pregnancy [35]. The regimens are based on azithromycin 1 g orally; amoxicillin is listed as an alternative therapy for CT for pregnant women at 500 mg orally three times daily for 7 days [31,36,37].

### 4.2. Epidemiology

The European Centre for Disease Prevention and Control (ECDC) reported 216,508 confirmed cases of CT in 27 EU/EEA countries, with a crude notification rate of 88 cases per 100,000 population. The notification rate is particularly high in women aged between 20 and 24 [38]. Between 1991 and 2021, 11,383 new cases of CT were reported in Italy. Of these, 3484 cases were reported in women. Considering the entire population, the largest number of cases was reported in the age group between 15 and 24 years. Regarding nationality, 20.3% of the total number of CT patients were foreigners (other European countries and Africa) [39]. In 2022, 70,881 cases of NG were notified in 28 European countries, with a crude notification rate of 17.9 per 100,000 population. Among women, the notification rate was 7.2 per 100,000 population (with 13,599 total cases). The largest proportion of cases reported was in the age group 25–34 years [40]. Between 1991 and 2021, 10,597 new NG cases were registered in Italy. Only 5.9% of cases (626 cases) were in women. In the total population of infected subjects, the highest number of cases was reported in subjects aged between 15 and 44 years. As for CT, infected foreigners (23.9% of the total number of cases) came from other European countries and Africa [39]. In a 2022 systematic review analyzing the global prevalence of NG infection in pregnant women, a higher incidence was confirmed in African countries (prevalence index 3.53). In Europe, a prevalence index of 0.52 was shown, with a prevalence of 0.1 in Italy [41]. In a recent study conducted in Brazil, a higher prevalence of CT than NG was found in pregnant women (9.9% vs. 0.6%) [42]. This difference was also found in sub-Saharan (CT 10.8% vs. NG 3.3%) [43] and in European countries (in the Netherlands, CT 1.8% vs. NG 0.4%) [44]. These data confirm what has already been shown in a study evaluating the global prevalence of STIs (CT 3.8% vs. NG 0.9%) [45].

### 4.3. Adherence to the New Intersociety Document

While our general data showed that an adequate number of women were screened for CT, a very low number were screened for NG. CT screening carried out using a molecular technique via swabbing (endocervical, vaginal, or urethral) [25] and NG screening by vaginal or endocervical swab must be offered to pregnant women with recognized risk factors at the first antenatal visit and during the third trimester [19]. The Italian intersociety position statement [1] is in line with both the ISS [19] and CDC guidelines [33]. In the CDC recommendations [46], the risk factors for STIs are: age < 25 years or age ≥ 25 years are at increased risk for infection (e.g., women aged ≥ 25 years who have a new sex partner, more than one sex partner, a sex partner with concurrent partners, or sex partner who has an STI) [47]. The risk factors determined by the new ISS guidelines [19] are illustrated in Table 1. In the intersociety position statement, originating from areas with a high incidence of STIs was also added as a risk factor [1]. As described above, in the medical records analyzed, there were only reported swabs performed in the third trimester. This confirms failure to adhere to the recommendations. The need for changes (from increased communication with physicians, to changes in the laboratory processes, etc.), was emphasized in a study carried out in the province of Alberta in Canada, where universal screening is recommended in the first trimester, and rescreening in the third trimester in those who resulted positive in the first one, and in those with risk factors. In fact, the authors demonstrated that although universal, screening in the first trimester was not performed on all women [48].

As can be seen from our data, screening in relation to risk factors was not properly followed. Considering that the average age in our hospital was in line with the average age of mothers presenting for childbirth in Italy (33.1 years among Italian women and 31.1 years among foreign citizens) [49], the number of women to be screened in relation to age only is low. Among women *≤* 25 years old (162), only 57 were screened for CT and none for NG. Concerning STIs, only 0.6% of the total number of women included in the study tested positive (diseases tested for included HIV, HBV, syphilis, and HCV). According to the results, in our center, the incidence of women with STIs was low, and those affected by STIs were not routinely screened for CT and NG.

Concerning the risk factor relating to women’s sexual history (Table 1), in Italy there is a cultural background whereby it is difficult for women to declare their sexual history. The questions suggested by the CDC [47] (e.g., “Are you currently having sex of any kind?” and “What kind of sexual contact do you have, or have you had?”) may have limited use in Italy.

In the new statement [1], maternal “origin” also accounts for a risk factor for STIs Our data (Table 5) indicated that CT swabs were performed in about 50% of women from countries with higher risk, whereas NG swabs were practically never performed. Furthermore, it must be considered whether the mother has resided for a long time in a country with a high incidence of CT or NG, shortly before or during pregnancy.

During the study period (February to August 2022), eye prophylaxis in Italy was not yet limited to specific groups of infants, consequentially all infants received antibiotic prophylaxis for the prevention of ON.

## 5. Conclusions

In this study, we observed that the risk factors for CT and NG screening during pregnancy are not being properly followed. This is highlighted by a remarkably low number of swabs performed for NG (only 13). Although the number of CT swabs is higher, pregnant women were not correctly tested as not all women under the age of 25 were screened, not all women with STIs were screened, and the nationality of the women was not taken into account. Although the number of women enrolled was limited, the sample represented approximately 0.6% of all the deliveries in Italy in 2022 [50]. Therefore, the results of our study may be representative of Italian reality. Properly following the guidelines for performing screening will lead to the prevention of negative outcomes related to CT and NG infection in pregnancy, including but not limited to ON.

## Figures and Tables

**Table 1:** Risk factors for STIs in pregnant women issued by the Italian Ministry of Health [19].

Parameter	Risk Factor
Age	<25 years
Sexual partner	New sexual partner (within 3 months)
	Multiple sexual partners Sexual partner with an STI
Sexual protection	Not using barrier protection properly in a non-reciprocal monogamous relationship
STIs	Having an STI
Sexual intercourse	Offering sexual intercourse in exchange for money or drugs

**Table 2:** General data: type of delivery, geographical nationality, gestational age of delivery, time of membrane rupture (pROM), maternal age (years), and number of women who received chlamydial or gonococcal swab. ΔS: deviation standard; ws: weeks; h: hours; ys: years.

**Delivery (n°, %)**	Vaginal delivery: 1243 (49.6%) Planned Cesarean section: 626 (25%) Urgent Cesarean section: 392 (15.6%) Operative vaginal delivery: 246 (9.8%)
**Geographical nationality (n°, %)**	Western Europe: 2155 (86%) Eastern Europe: 181 (7.2%) South Asia: 45 (1.8%) Central and South America: 39 (1.6%) North Africa: 31 (1.2%) Remaining Africa: 29 (1.2%) South-East Asia and the Far East: 13 (0.5%) North America: 6 (0.2%) Russia and Central Asia: 6 (0.2%) Middle East: 2 (0.1%)
**Gestational Age (ws ± ΔS)**	39.2 ± 1.2
**pROM (h ± ΔS)**	3.8 ± 5.6
**Maternal age (ys ± ΔS)**	33.6 ± 5.4
**Chlamydial swab (n°, %)**	Negative: 1055 (42.1%) Positive: 7 (0.3%) Not screened: 1445 (57.6%)
**Gonococcal swab (n°, %)**	Negative: 13 (0.5%) Positive: 0 Not screened: 2494 (99.5%)

**Table 3:** Comparison of mean gestational age (weeks), time of membrane rupture (pROM) (hours), and maternal age (years) in groups of women who had or had not been screened for chlamydia or gonococcus. ΔS: deviation standard.

	Gestational Age (Weeks, Median ± ΔS)	*p*	Prom (Hours, Median ± ΔS)	*p*	Mother Age (Years, Median ± ΔS)	*p*
**CHLAMYDIAL SWAB**						
*Not screened (1445)*	39.2 ± 1.2	0.05	4 ± 5.8	0.07	33.6 ± 5.5	0.18
*Screened (1062)*	39.1 ± 1.3		3.6 ± 5.3		33.8 ± 5.3	
**GONOCOCCAL SWAB**						
*Not screened (2494)*	39.2 ± 1.2	0.80	3.8 ± 5.6	0.5	33.7 ± 5.4	0.93
*Screened (13)*	39.1 ± 1.5		2.6 ± 3.8		34 ± 4.8	

**Table 4:** Comparison of delivery types and screening.

DELIVERY						
	Vaginal Delivery	Planned Cesarean Section	Urgent Cesarean Section	Operative Vaginal Delivery	Total	*p*
**CHLAMYDIAL SWAB**						0.294
*Screened*	540 (21.5%)	267 (10.7%)	149 (5.9%)	106 (4.2%)	1062 (42.4%)	
*Not screened*	703 (28%)	359 (14.3%)	243 (9.7%)	140 (5.6%)	1445 (57.6%)	
TOTAL	1243 (49.5%)	626 (25%)	392 (15.6%)	246 (9.8%)	2507 (100%)	
**GONOCOCCAL SWAB**						0.988
*Screened*	7 (0.3%)	3 (0.1%)	2 (0.1%)	1 (0.1%)	13 (0.6%)	
*Not screened*	1236 (49.2%)	623 (24.9%)	390 (15.5%)	245 (9.7%)	2494 (99.4%)	
TOTAL	1243 (49.5%)	626 (25%)	392 (15.6%)	246 (9.8%)	2507 (100%)	

**Table 5:** Distribution based on maternal nationality.

	NATIONALITY (DIVIDED INTO GEOGRAPHIC AREAS)											
	Western Europe	Eastern Europe	North Africa	Central and South Africa	North America	Central and South America	Middle East	Russia and Central Asia	South-East Asia and Far East	South Asia	Total	*p*
**CHLAMYDIAL SWAB**												
*Screened*	897 (35.8%)	80 (3.2%)	15 (0.6%)	15 (0.6%)	2 (0.1%)	23 (0.9%)	2 (0.1%)	1 (0.1%)	4 (0.2%)	23 (0.9%)	1062 (42.4%)	0.14
*Not screened*	1258 (50.1%)	101 (4%)	16 (0.6%)	14 (0.6%)	4 (0.2%)	16 (0.6%)	0	5 (0.2%)	9 (0.4%)	22 (0.9%)	1445 (57.6%)	
TOTAL	2155 (85.9%)	181 (7.2%)	31 (1.2%)	29 (1.2%)	6 (0.3%)	39 (1.5%)	2 (0.1%)	6 (0.3%	13 (0.6%)	45 (1.8%)	2507 (100%)	
**GONOCOCCAL SWAB**												
*Screened*	11 (0.4%)	1 (0.1%)	0	0	0	0	0	1 (0.1%)	0	0	13 (0.6%)	<0.001
*Not screened*	2144 (85.2%)	180 (7.1%)	31 (1.2%)	29 (1.2%)	6 (0.2%)	39 (1.6%)	2 (0.1%)	5 (0.2%)	13 (0.6%)	45 (1.8%)	2494 (99.5%)	
TOTAL	2155 (85.6%)	181 (7.2%)	31 (1.2%)	29 (1.2%)	6 (0.2%)	39 (1.6%)	2 (0.1%)	6 (0.3%)	13 (0.6%)	45 (1.8%)	2507 (100%)	

**Table 6:** Distribution of women screened or not screened according to STI status and age. Yo: years old.

**CHLAMYDIAL SWAB**			
	***Screened***	***Not* *Screened***	***p***
*STI-positive*	4 (0.2%)	6 (0.3%)	0.876
*STI-negative*	1058 (42.2%)	1439 (57.3%)	
*>25 yo*	1005 (40%)	1340 (53.4%)	0.05
*≤25 yo*	57 (2.3%)	105 (4.3%)	
**GONOCOCCAL SWAB**			
	***Screened***	***Not* *Screened***	***p***
*STI-positive*	0	10 (0.4%)	0.81
*STI-negative*	13 (0.6%)	2484 (99%)	
*>25 yo*	13 (0.6%)	162 (6.5%)	0.34
*≤25 yo*	0	2332 (92.9%)	

**Table 7:** Distribution of negative or positive results for CT or NG conducted in women with STIs and distribution of negative or positive results for CT or NG conducted in relation to maternal age. Yo: years old.

**CHLAMYDIAL SWAB**			
	***Negative***	***Positive***	***p***
*HBV*	3	0	not evaluable
*HIV*	1	0	
*LUE*	0	0	
**CHLAMYDIAL SWAB**			
	***Negative***	***Positive***	***p***
*>25 yo*	999	6	0.29
*≤25 yo*	56	1	
**GONOCOCCAL SWAB**			
	***Negative***	***Positive***	***p***
*>25 yo*	0	0	not evaluable
*≤25 yo*	13	0	

## Data Availability

The data that support the findings of this study are available from the corresponding author, Vito Mondì, upon reasonable request.

## Supplementary Materials

The following supporting information can be downloaded at: https://www.mdpi.com/article/10.3390/pathogens13070570/s1, File S1: List of the countries subdivided in geographical area.
