# Supplementary material for: Are Chlamydia Trachomatis and Neisseria Gonorrhoeae Screenings in Pregnant Women Being Properly Performed? A Single-Center Retrospective Observational Study in Italy
Source: Pathogens. 2024 Jul 8;13(7):570. doi: 10.3390/pathogens13070570 (PMC11279639; doi:10.3390/pathogens13070570)
Supplement: Supplementary file 1 [file pathogens-13-00570-s001.zip › pathogens-3054475-supplementary.pdf]

Supplementary File S1

List of the country subdivided in geographical area. The country cited in the list represent the nationality of the women near the childbirth admitted in our hospital from February to August 2022.

Western Europe	Italy, United Kingdom, Spain, Germany, Sweden, Austria, France, Switzerland, Belgium
Eastern Europe	Romania, Bulgaria, Poland, Turkey, Albania, Republic of Macedonia, Kosovo, Serbia, Hungary, Slovakia
Russia and Central Asia	Russia, Moldova, Ukraine, Belarus, Georgia
Middle East	Israel
South Asia	Afghanistan, Iran, Pakistan, India, Bangladesh, Nepal, Sri Lanka
South-East Asia and the Far East	Thailand, China, Philippine
North Africa	Morocco, Egypt, Algeria, Tunisia,
Remaining Africa	Nigeria, Ethiopia, Senegal, Guinea, Ivory Coast, Congo
North America	Unites States of America
Central and South America	Cuba, Ecuador, El Salvador, Peru, Cile, Brazil, Honduras, Colombia, Dominican Republic, Venezuela, Haiti
